# Proton Beam Therapy in Gynecological Cancers: A Systematic Review of Indications, Complications, and Limitations

**DOI:** 10.3390/medicina62020334

**Published:** 2026-02-06

**Authors:** Vito Andrea Capozzi, Giulia Martignon, Elisa Scarpelli, Alessandra De Finis, Stefano Restaino, Giuseppe Vizzielli, Roberto Berretta

**Affiliations:** 1 Department of Medicine and Surgery, University Hospital of Parma, 43126 Parma, Italy; 2 Department of Medicine and Surgery, Doctoral School in Health Sciences and Technology, University of Bologna, 40126 Bologna, Italy; 3 Clinic of Obstetrics and Gynecology, “Santa Maria della Misericordia” University Hospital, Azienda Sanitaria Universitaria Friuli Centrale, 33100 Udine, Italy; 4 Medical Area Department (DAME), University of Udine, 33100 Udine, Italy

**Keywords:** proton beam therapy, gynecological cancer, reirradiation

## Abstract

*Background and Objectives*: Gynecological cancers frequently require radiation therapy (RT) in primary, adjuvant, or salvage settings. However, photon-based RT is associated with non-negligible toxicity, and treatment of pelvic recurrences after prior irradiation remains challenging. Proton beam therapy (PBT), due to its favorable dose distribution and reduced exposure of organs at risk (OARs), has emerged as a potential alternative, particularly in re-irradiation scenarios. Despite its expanding use in other malignancies, evidence supporting PBT in gynecologic cancers remains limited. This systematic review aims to investigate the use of PBT in gynecological cancers and its associated complications. *Materials and Methods*: This systematic review was conducted according to PRISMA guidelines and registered in PROSPERO. A comprehensive search (2000–2025) identified studies investigating PBT in gynecologic cancers. Eligible designs included randomized trials and prospective and retrospective series. Reported adverse events were categorized as GI, GU, or other, and only grade ≥3 CT-CAE complications were considered. *Results*: Of 580 records screened, 9 studies comprising 232 patients met inclusion criteria. Most patients were treated for endometrial (n = 147) or cervical (n = 75) cancer; 90 received chemotherapy. Overall, severe toxicity occurred in 15.2% of patients. GI complications ranged from 0–14% and GU from 0–33%. Complication rates were lowest in adjuvant or de novo treatment series (0–10%), whereas re-irradiation cohorts showed higher rates (up to 33% GU). Comparative studies suggested a possible advantage of PBT over IMRT, particularly for GI toxicity, though data remain limited. *Conclusions*: Severe GI and GU toxicity after PBT in gynecologic cancers appears infrequent, particularly in primary and adjuvant settings, though re-irradiation remains challenging. Current evidence is restricted to small and heterogeneous studies. Ongoing phase II trials will provide prospective data to clarify feasibility, toxicity, and long-term outcomes. Until then, PBT in gynecologic oncology should be regarded as investigational.

## 1. Introduction

Gynecological cancers affect nearly 1.4 million women worldwide each year, with cervical and endometrial cancers ranking as the second and sixth most common malignancies in women, respectively [1]. Radiation therapy (RT), traditionally delivered with photon-based external beam radiotherapy (EBRT), plays a prominent role in the treatment of gynecological malignancies, either as an exclusive or in combination with chemotherapy [2,3,4,5]. RT is used as an adjuvant treatment after hysterectomy for endometrial cancer, if risk factors for recurrence are present, or as an adjuvant or definitive treatment for cervical cancer, either after radical hysterectomy or for unresectable or advanced-stage disease, respectively [2,3]. Finally, RT is also used for primary and/or recurrent vulvar and vaginal cancer [4,5].

Whether considering EBRT or brachytherapy (BRT), RT is essential for local disease control but has a proven non-negligible complication rate [6,7,8]. Nevertheless, recurrence after primary RT remains a major therapeutic challenge.

Over the years, the only option for central pelvic recurrence was pelvic exenteration (PE). However, this procedure, first described in 1948, is extremely complex, not always feasible due to the patient’s performance status, and is burdened by high intraoperative morbidity and mortality [9,10,11,12].

In this context, secondary radiotherapy has gained increasing interest, with efforts directed toward minimizing radiation exposure to organs at risk (OARs) [13]. Technological innovations such as intensity-modulated radiation therapy (IMRT) and volumetric modulated arc therapy (VMAT) have significantly improved dose conformity and reduced toxicity to surrounding tissues induced by photon-based RT [14,15,16,17]. Finally, these technological advances led to the development of proton beam therapy (PBT), which uses high-energy particles instead of photons [18]. Indeed, protons release the majority of their energy at a defined depth, known as the Bragg peak, with minimal exit dose beyond the target [19]. This characteristic allows for improved sparing of adjacent OARs compared with photon techniques, resulting in fewer side effects.

Although PBT has been investigated across different cancers and is an established standard of care in selected contexts such as pediatric malignancies and sarcomas, its role in gynecological cancers remains investigational, with current evidence largely limited to dosimetric analyses and small clinical series [20,21,22,23,24,25,26].

To date, given the high rate of pelvic recurrences after primary RT and the morbidity of salvage surgery, re-irradiation represents a clinically relevant field where PBT could offer a therapeutic alternative, also in gynecological disease. This systematic review aims to investigate the application of PBT in recurrent gynecological cancers, focusing on indications, complications, and limitations.

## 2. Materials and Methods

The review adhered to the PRISMA (Preferred Reporting Items for Systematic Reviews and Meta-Analyses) guidelines and was registered in the PROSPERO database with registration number CRD420251111158. The following keywords have been selected: “proton beam therapy and gynecological cancer”, “proton therapy and gynecology”, “proton beam radiotherapy and gynecology”, “reirradiation and gynecological cancer”. The literature search was independently conducted by two authors in PubMed/MEDLINE, Scopus, Embase, and Web of Science. The search was last updated on 10 March 2025. Two authors reviewed the selection for consistency (VAC and ES). Articles about proton beam therapy for treating gynecological cancers were included, regardless of the primary site or the timing of the treatment (adjuvant, exclusive primary treatment, or secondary treatment). Studies not evaluating PBT complications were excluded.

Articles published between 2000 and 2025 were screened. Randomized controlled trials, case series, retrospective, and prospective studies in the English language were included. Adverse events were categorized as gastrointestinal (GI) or genitourinary (GU). All other events were grouped under the category ‘other complications’. Complications were grouped according to the Common Terminology Criteria for Adverse Events (CT-CAE) [27]. Only grade ≥ 3 CT-CAE complications were included, corresponding to severe or medically significant adverse effects that are not immediately life-threatening but require active medical intervention or substantially impair daily functioning. The authors independently screened all abstracts and then evaluated the full-length text of eligible articles to extract relevant data. Two additional authors (RB and VAC) discussed and mediated any discrepancies to reach a consensus. All references were analyzed to evaluate additional eligible studies. The researchers reached an agreement about potential relevance by consensus and according to PRISMA statement guidelines [28]. Studies not aligning with the purpose of the study, case reports, redundant studies, abstracts, and articles not in the English language were excluded. Risk of bias was assessed independently by two reviewers (GM and ES) using the Joanna Briggs Institute (JBI) Critical Appraisal Checklists for cohort studies [29]. Each item was rated as Yes, No, Unclear, or Not Applicable. Disagreements were resolved by consensus. No numerical scores were calculated; overall risk of bias was determined qualitatively based on the distribution of item-level judgments. The risk was considered low when fewer than three domains were rated as “unclear” and no domain as “no.” It was considered moderate when at least three domains were rated as “unclear” and up to one domain as “no.” Studies with two or more domains rated as “no” were classified as having a high risk of bias.

## 3. Results

**Figure 1:**
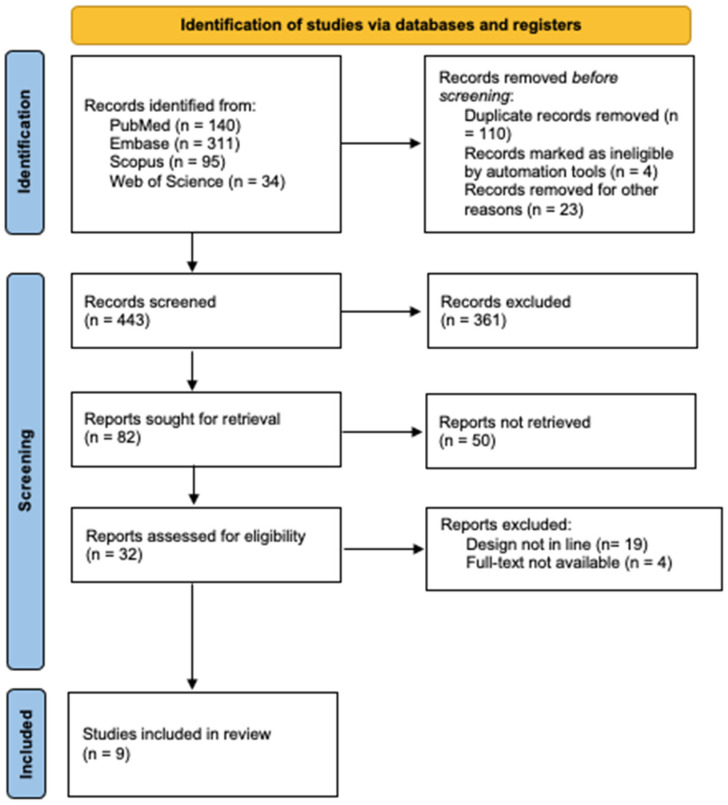
PRISMA flow diagram.

A total of 580 articles published between 2000 and 2025 were identified from the primary database search. After screening, 548 articles were excluded. Finally, 32 articles were considered eligible for the systematic review; of these, 23 were also excluded because the study design was not in line with the aim of the review, or the full text was not available. Overall, 9 studies were finally selected for this review: 4 prospective studies [30,31,32,33], 4 retrospective studies [6,34,35,36], and 1 case series [37]. The selection process is summarized in the PRISMA flowchart (Figure 1).

The nine studies encompassed 232 patients (Table 1). Most were treated for endometrial cancer (n. 147), followed by cervical (n. 75), vaginal (n. 2) and vulvar (n. 1) cancer, while tumor type was not specified for seven patients. Ninety-two patients received chemotherapy (concurrent, sequential, or adjuvant). Thirty-five patients had history of previous photon radiation treatment, while 197 patients were naïve to radiotherapy. One study [6] took Patients-Related Outcomes version of the CTCAE (PRO-CTCAE) into account for evaluating the complication rates and was excluded from the cumulative analysis [38]. One study [31] investigated 27 patients who underwent combined photon and proton beam therapy. Two studies [6,36] compared the complication rate between patients treated with PBT and RT.

Overall, the incidence of severe (grade ≥ 3 CT-CAE) complications after PBT was 15.2% (n = 25/165). Reported rates varied between 0% [30] and 14% [33] for GI, and between 0% and 33% [37] for GU toxicity. In comparative analyses, the PRO-CTCAE study [6] found total G3 or higher complication rates of 77% for PBT and 87% for IMRT (GU 23% vs. 36%, GI 9% vs. 33%), whereas the CTCAE-based study [36] reported complication rates of 0% for PBT and 16% for IMRT, with no grade ≥3 GI or GU events in either group.

Based on the JBI Critical Appraisal Checklists, among the nine studies, one [30] was judged at low risk of bias, five [6,32,33,35,36] at moderate risk, and three [31,34,37] at high risk of bias (See Supplementary Materials). The main limitations identified across studies were the absence of control groups in single-arm cohorts, incomplete adjustment for confounding factors, and short or variably reported follow-up durations. Statistical methods were generally appropriate in prospective and comparative studies, but less rigorous or purely descriptive in retrospective case series such as Berlin et al. [34]. Only one study reported a formal prospective design with predefined endpoints and complete follow-up, which contributed to its classification as low risk of bias.

## 4. Discussion

### 4.1. Key Findings of the Review

Across the nine studies included, the overall rate of grade ≥3 CT-CAE complications after PBT was 15.2% (n = 25/165), with a wide variability among series (0–49.6%), which is in line with toxicities from photon-based radiotherapy, ranging approximately from 10 to 20%, depending on disease stage, treatment volume, and use of concurrent chemotherapy [7]. Differences in reported toxicity largely reflected the diversity of patient populations, treatment intent, and outcome assessment methods. The study by Anderson et al. [6], which uniquely used PRO-CTCAE, found an overall toxicity rate of 77% for any grade and a significantly lower rate of GI in PBT compared to IMRT (frequent diarrhea in 9% vs. 33%, *p* = 0.05). However, due to methodological heterogeneity in toxicity grading, this study was excluded from pooled estimates but included in the qualitative synthesis. The pattern and frequency of toxicity differed substantially depending on whether patients had received prior pelvic irradiation. Among patients without previous radiotherapy, treated either in the adjuvant or primary setting, severe complications were uncommon, with an overall rate of 11.5% (10/130) [30,31,32,33,34,36]. In Russo et al., the rate of acute grade 3 toxicity reached 38%, mainly gastrointestinal, which the authors attributed to the extensive use of extended-field irradiation (81% of patients) and concurrent chemotherapy rather than to PBT itself [33]. Taken together, these studies indicate that in radiotherapy-naïve patients, PBT is feasible and generally well tolerated, with severe toxicity typically below 15%.

In contrast, patients undergoing re-irradiation experienced higher toxicity rates. Pollock et al. documented severe complications in 24% of cases (7/29), including two grade 3 gastrointestinal events (acute diarrhea and late rectal hemorrhage) [31]. Mizuno et al. reported grade 3–4 events in 33% of a small cohort (n = 6) previously treated with photons, consisting of two cases of radiation-induced cystitis with hematuria and one bowel perforation, which occurred outside the treatment field and was attributed to disease progression [37]. These findings underscore that, although PBT offers improved dose conformity and organ sparing compared with photons, re-irradiation remains a high-risk scenario because of cumulative dose constraints to bowel and bladder.

### 4.2. Heterogeneity and Methodological Limitations

The main limitations of the present study lie in the substantial clinical and methodological heterogeneity of the studies and patient populations undergoing PBT. Most of the studies included de novo cases, postoperative settings, and recurrences after prior RT. Chemotherapy was administered inconsistently, either sequentially or concurrently, which may have contributed to hematologic rather than GI or GU toxicity. Moreover, toxicity assessment was not uniform, with some studies reporting adverse events according to CTCAE [27], while others used PRO-CTCAE [38], limiting comparability across series. Sample sizes were generally small, often single-center, with short follow-up periods. In addition, the restriction to peer-reviewed studies published in the English language may have led to the exclusion of potentially relevant data. Importantly, no randomized trials have been conducted to date, and the risk of selection bias is high. Collectively, these limitations preclude any causal inference regarding a toxicity advantage of PBT over modern photon-based techniques.

### 4.3. Indications to Proton Therapy and Current Standards

PBT has already been incorporated as standard of care in specific settings. In pediatric malignancies, its ability to reduce the integral dose translates into a lower risk of growth impairment and second cancers [39,40]. In skull base sarcomas, the steep dose gradients achievable with protons allow tumor control while sparing critical structures such as the brainstem and optic apparatus [41]. In other tumor types, including breast (RADCOMP trial) [42], esophageal [43], and head-and-neck cancers [44], large randomized or prospective phase II trials are ongoing. These aim to clarify whether dosimetric advantages translate into clinically meaningful reductions in toxicity. For gynecologic malignancies, radiotherapy, delivered with IMRT or VMAT and combined with brachytherapy when indicated, remains the standard of care according to international guidelines [2,3,4,5]. Importantly, all pivotal randomized trials that underpin these recommendations were conducted using photon-based techniques [45,46]. Proton beam therapy is therefore not specifically endorsed in current guidelines, reflecting the very limited clinical evidence available in gynecologic oncology.

The implementation of proton therapy is not determined solely by clinical evidence, but also by national reimbursement frameworks and cost considerations, which vary substantially across healthcare systems. In the United States, the 2023 ASTRO Model Policy expanded approved indications to include selected thoracic, abdominal, and pelvic tumors, but gynecologic cancers are not explicitly listed [47]. In Italy, the 2021 report of the Istituto Superiore di Sanità explicitly includes proton therapy among the ten conditions reimbursed within the national health service, particularly for re-irradiation [48]. Within this framework, the document acknowledges the potential role of protons for pelvic recurrences of gynecologic tumors. International consensus statements (ESTRO/EORTC) on re-irradiation recommend the use of highly conformal techniques with cumulative dose assessment, a context in which PBT may be considered if photon plans exceed tolerance [49]. In fact, a recognized limitation of IMRT and VMAT is the ‘low-dose bath,’ in which large volumes of surrounding normal tissues are exposed to low and intermediate doses as a consequence of multiple beam angles. This phenomenon contributes to late toxicity and represents a major constraint of photon-based approaches.

In summary, these guidelines and policy positions underscore that, while PBT is established or under active evaluation in several malignancies, in gynecologic cancers, it remains investigational. PBT use should therefore be limited to clinical trials or highly selected cases, particularly in re-irradiation scenarios where OAR sparing cannot be achieved with photon techniques.

### 4.4. Contraindications

The contraindications for proton therapy are the same as those for traditional photon radiotherapy. General contraindications include pregnancy, certain autoimmune or connective tissue disorders, and prior radiation to the same area. While the harmful effects of radiation exposure on the fetus are well known [50,51], the presence of collagen vascular disease (CVD) and inflammatory bowel disease (IBD) is considered a radiation oncology dogma rather than an actual contraindication. In fact, a meta-analysis of 621 patients [52] indicates that the risk of grade 4 or 5 complications in patients with these conditions is minimal, <5% and <1%, respectively. Finally, about previous radiation treatment, a growing body of evidence indicates a strong dose-volume relationship in the development of bowel toxicity [53,54]. Due to its intrinsic characteristics, proton beam therapy permits only a little or no scattered or exit radiation outside the tumor target, reducing the dose to the OARs [18]. As mentioned above, this review observed higher complication rates in patients with previous photon radiation treatment [37], though they achieved optimal local disease control. Further studies analyzing the toxicity of re-irradiation in patients previously treated with PBT would better clarify the potential of this technology. In the event of cancer recurrence, re-irradiation should not be considered a contraindication, but rather a condition specific to the patient that requires personalized treatment planning.

### 4.5. Potential Applications in Gynecologic Oncology and Limitations

In gynecologic oncology, several clinical scenarios highlight the potential relevance of PBT. Pelvic recurrences after prior irradiation represent one of the most challenging settings. Pelvic exenteration, still considered the reference salvage option, achieves 5-year survival rates of only 30–40% in carefully selected patients, with perioperative mortality of 5–10% [10,55]. Photon re-irradiation is feasible but constrained by bowel and bladder tolerance. A second clinically relevant scenario is extended-field irradiation for para-aortic nodal involvement, which occurs in about 10–25% of locally advanced cervical cancers and 5–15% of high-risk endometrial cancers [56,57,58]. Photon techniques expose large volumes of small bowel, kidneys, and pelvic bone marrow to low and intermediate doses. In contrast, PBT has been shown to lower the dose to normal tissues, resulting in reduced toxicity [25,59].

Finally, the issue of long-term survivorship is critical. Many women with cervical or endometrial cancer are relatively young, and late toxicities have profound consequences on quality of life. Reported chronic complications after pelvic RT include enteritis, cystitis, fistulae, and bowel obstruction, with grade ≥3 events occurring in approximately 10–20% of patients [7]. A contributing factor is the so-called “low-dose bath”, characteristic of IMRT and VMAT, in which large volumes of normal tissue are exposed to low and intermediate doses. This phenomenon has been associated with increased risks of chronic GI/GU toxicity and secondary malignancies. By reducing low-dose exposure, PBT may mitigate these long-term effects.

Despite the clear dosimetric rationale for PBT, several factors explain why PBT has not yet become standard in gynecologic oncology. First, the clinical scenarios where PBT may provide the greatest benefit, such as pelvic re-irradiation or extended-field irradiation, are relatively uncommon, making it difficult to accrue large patient numbers for prospective studies. Second, gynecologic cancers represent a heterogeneous group of diseases with different primary sites, treatment intents (adjuvant, definitive, or salvage), and combinations with chemotherapy, which complicates the design of uniform clinical trials. Third, modern photon-based techniques such as IMRT and VMAT already achieve highly conformal dose distributions and are widely available, raising the evidentiary bar for demonstrating added clinical value with protons [60]. Moreover, the high cost and limited accessibility of proton centers restrict trial participation and patient referral, as underscored by health policy documents that continue to categorize PBT for gynecologic cancers as investigational [47,48]. Furthermore, many of the expected advantages of PBT pertain to late toxicity reduction, which requires long-term follow-up to be convincingly demonstrated. Together, these barriers contribute to the paucity of randomized data and explain why, in contrast to pediatric tumors or skull base sarcomas, where prospective evidence has been practice-changing, PBT remains investigational in gynecologic oncology.

Finally, no specific contraindications to PBT have been reported compared with conventional photon-based radiotherapy. Patient selection criteria largely overlap, being mainly determined by performance status, disease extent, and organ tolerance [2,3,49]. While dosimetric data suggest reduced exposure of organs at risk with PBT, the general contraindications and clinical precautions remain the same as for modern photon techniques such as IMRT or VMAT.

In line with previously discussed evidence and recommendations, a summary of clinical scenarios where PBT may or should be considered is presented in Table 2.

### 4.6. Future Perspectives and Ongoing Trials

Evidence on PBT for gynecologic malignancies is still scarce, but several prospective efforts are now underway to better define its role. These studies are primarily designed to assess feasibility, dosimetric advantages, and treatment-related toxicity, rather than oncologic efficacy or survival outcomes. The APROVE trial was among the first prospective studies, demonstrating the feasibility and tolerability of postoperative PBT for cervical and endometrial cancer [30]. Building on this, two phase II trials are currently recruiting. The PROPS GYN trial (NCT05758688) is a US multicenter study evaluating adjuvant whole-pelvis PBT after hysterectomy for endometrial or cervical cancer, with clinician-reported acute GI toxicity as its primary endpoint, reflecting a feasibility- and toxicity-oriented study design. The expected completion date is 2026 [61]. In Europe, the PROTECT trial (NCT05406856) is a non-randomized phase II study comparing adaptive IMPT with IMRT/VMAT in locally advanced cervical cancer. Its primary focus is pelvic bone marrow and bowel dose reduction, while secondary endpoints include quality of life, immune response, and safety. The trial is ongoing and scheduled to be completed in 2026 [62]. Earlier studies, such as NCT01019278, which tested the feasibility of combining PBT with cisplatin for cervical cancer with para-aortic nodal involvement, and NCT01600040, a pilot trial of post-hysterectomy PBT, were registered more than a decade ago but had limited accrual, and no updates are available on current status [63,64]. Overall, these ongoing trials are expected to provide critical prospective data on toxicity, clinical outcomes, and quality of life. Until such data are available, the use of PBT in gynecologic oncology should be regarded as investigational and limited to clinical trials or selected patients in specialized centers.

## 5. Conclusions

This systematic review shows that grade ≥3 GI and GU toxicity after PBT for gynecologic malignancies occurs in about 15% of patients, with GI events being more frequent. Toxicity was low in adjuvant and primary settings, but higher in re-irradiation, though still lower than with photon-based re-treatment. Limited comparative data suggest a potential advantage of PBT over IMRT, especially for GI toxicity. Current evidence is restricted to small, heterogeneous series without randomized data. Ongoing prospective studies such as PROPS-GYN, and PROTECT, mainly designed to assess feasibility and dosimetric outcomes, will lay the groundwork for well-designed randomized trials capable of providing solid evidence on late toxicity and oncologic outcomes in gynecologic oncology.

## Figures and Tables

**Table 1:** Articles included in the review.

Study, Year	Type of Study	Patients n. and Type of Treatment	Mean Age, y	Pathology, Treatment Setting	Previous Radiotherapy with Photons	Delivered Dose (Gy)	Concurrent Brachytherapy (Patients %), Delivered Dose	Para-Aortic Fields, Patients %	Chemotherapy, n. Patients	Follow-Up (Months)	Severe Complications (G3 or >) Rate	Method Used
Anderson et al., 2022 [6]	Retrospective study	67 - 22 PBT - 45 IMRT	67.4 PBT group 72.7 IMRT group 64.8	Endometrial cancer - PBT group 19 primary treatment 3 recurrent disease - IMRT group 45 primary treatment	No	PBT 45–50.4 IMRT 45–50.4	PBT group 76%, 10 Gy 2 fr. IMRT group 78%, 15 Gy 3 fr.	PBT group 14% IMRT group 33%	PBT group 0/22 IMRT group 2/45	12	Any G3 or >: - PBT group 17/22 (77%) - MRT group 39/45 (87%) GU G3 or >: - PBT group 5/22 (23%) - IMRT group 16/45 (36%) GI G3 or >: - PBT group 2/22 (9%) - IMRT group 15/45 (33%)	PRO-CTCAE
Arians et al., 2023 [28]	Prospective study	25 PBT	64	17 endometrial cancer 8 cervical cancer 25 post-operative primary setting	No	45–50.4 (RBE)	100% 5 Gy 2 fr	4%	Endometrial cancer 13/17 Cervical cancer 7/8	24	Any G3 or >: 0	CT-CAE
Berlin et al., 2022 [34]	Retrospective study	23 PBT	59	12 endometrial cancer 10 cervical cancer 1 vaginal cancer 20 postoperative primary setting 3 definitive chemoradiation	No	50.4 Gy median	43.5%	34.8%	Any chemotherapy 22/23	Median 57.8 (95% CI 27.7–77.8)	GI G3 1/23 (4.3%)	CT-CAE
Kagei et al., 2003 [29]	Prospective study	25 PBT	62	25 Cervical cancer (locally advanced) Primary treatment	No	PBT median 61 Gy 3–4 fr. IMRT 50.4 Gy 28 fr.	/	/	/	139 (11–184)	GU G4 1/25 (4%) GI G4 1/25 (4%)	CT-CAE
Mizuno et al., 2025 [35]	Case series	6 PBT	55.5	1 Endometrial cancer 4 Cervical cancer 1 Vulvar cancer Treatment of the para-aortic recurrence	Yes	50–60 Gy (RBE) Boost 6–10 Gy (RBE)	/	/	Concurrent chemotherapy 3/6 (50%)	N.A.	Any G3 or >: 5/6 (83.3%) GU G3: 2/6 (33%) GI G4: 1/6 (16.6%)	CT-CAE
Pollock et al., 2023 [33]	Retrospective study	29 PBT	65	25 recurrent gynecologic cancer (15 endometrial cancer) 4 de novo gynecologic cancer but previous RT	Yes 100% partial overlap 52% complete target overlap	Median 49.2 Gy	20% /	/	/	Median 23	Any G3 or >: 7/29 (24%) GI G3 2/29 (6.9%): - 1 acute - 1 late GU G30	CT-CAE
Wark et al., 2024 [34]	Retrospective study	50 - 25 PBT - 25 VMAT	61	PBT group 8 cervical cancer 17 endometrial cancer VMAT group 8 cervical cancer 17 endometrial cancer	No	PBT group - 9/25 45 Gy (RBE) - 16/25 50.4 Gy (RBE) VMAT group - 9/25 45 Gy (RBE) - 16/25 50.4 Gy (RBE)	100%, /	PBT 4% VMAT 4%	PBT group 25/25 - 8/25 Previous CT - 7/25 Concurrent CT - 5/25 Adjuvant CT VMAT group 17/25 - 8/25 Previous CT - 7/25 Concurrent CT - 2/25 Adjuvant CT	24 (PBT) N.A. (VMAT)	Any G3 or >: - PBT 0/25 (0%) - VMAT 4/25 (16%) GU G3 or >: - PBT 0 - VMAT 0 GI G3 or >: - PBT 0 - VMAT 0	CT-CAE
Lin et al., 2016 [30]	Prospective study	11 PBT	55	7 cervical cancer 2 endometrial cancer Primary treatment post-hysterectomy 1 recurrent endometrial cancer 1 vaginal cancer (previous hysterectomy for benign condition)	No	45–50.4 Gy (RBE)	45.5%	/	Any CT 11/11 - 7/11 Concurrent CT - 2/11 Sandwich CT - 2/11 Both sandwich CT and concurrent CT	N.A.	Any G3 or >: 2/11 (18.2%) GI G3: 1/11 (9.1%) GU G3: 0	CT-CAE
Russo et al., 2025 [31]	Prospective study	21 PBT	59.7	15 stage IIIC uterine cancer 6 N1 cervical cancer Primary treatment	No	Median dose 45 Gy (RBE)	95%	81%	Any CT 17/21 - 11/21 sequential CT - 6/21 concurrent CT	60	Any acute G3 8/21 (38%) Acute GI G3 3/21 (14%) Acute GU G3 0/21 Any late G3 2/21 (9.5%) Late GI G3 1/21 (4.7%) Late GU G3 0/21	CT-CAE

Abbreviations: PBT: Proton Beam Therapy; IMRT: Intensity-Modulated Radiation Therapy; VMAT: Volumetric Arc Therapy; BRT: Brachytherapy; GU: Genitourinary; GI: Gastrointestinal; RBE: Relative Biological Effect; CT: Chemotherapy; CT-CAE: Common Terminology Criteria for Adverse Events; PRO-CTCAE: Patients-Related Outcomes-CTCAE; N.A.: Not available.

**Table 2:** Clinical scenarios and role of proton beam therapy relative to current standards.

Clinical Scenario	Role of PBT Relative to Current Standards	Rationale
Pediatric malignancies	Standard of care	Reduction in integral dose and risk of growth impairment and secondary malignancies
Skull base sarcomas	Standard of care	High dose conformity and sparing of critical structures
Pelvic recurrence after prior radiotherapy (gynecologic cancers)	Accepted option in selected cases	Need to limit cumulative dose to bowel, bladder, ureters, pelvic nerves
Para-aortic nodal irradiation (gynecologic cancers)	Investigational/ selected cases	Potential reduction in dose to kidneys, bowel, and bone marrow
Extended-field pelvic irradiation	Investigational	Reduction in low- and intermediate-dose exposure to organs at risk
Primary/adjuvant treatment of gynecologic cancers	Investigational	Limited clinical evidence; photon-based RT remains standard

## Data Availability

No new data were created or analyzed in this study. Data sharing is not applicable to this article.

## Supplementary Materials

The following supporting information can be downloaded at: https://www.mdpi.com/article/10.3390/medicina62020334/s1, File S1. JBI Critical Appraisal Checklist for Cohort Studies. File S2. PRISMA_2020_checklist.
