# Supplementary material for: Proton Beam Therapy in Gynecological Cancers: A Systematic Review of Indications, Complications, and Limitations
Source: Medicina (Kaunas). 2026 Feb 6;62(2):334. doi: 10.3390/medicina62020334 (PMC12941855; doi:10.3390/medicina62020334)
Supplement: Supplementary file 1 [file medicina-62-00334-s001.zip › File S1. Suppl Mat_Cohort Studies_Risk of bias assessment.pdf]

JBI CRITICAL APPRAISAL CHECKLIST FOR COHORT STUDIES

	Q1	Q2	Q3	Q4	Q5	Q6	Q7	Q8	Q9	Q10	Q11	Risk of bias
Arians et al., 2023	⚪	⚪	🟢	🟡	🟡	🟢	🟢	🟢	🟢	🟢	🟢	Low
Berlin et al., 2023	⚪	⚪	🟢	🔴	🔴	🟢	🟢	🟡	🟢	🟢	🔴	High
Kagei et al., 2003	⚪	⚪	🟢	🔴	🔴	🟢	🟡	🟢	🟢	🟢	🔴	High
Mizuno et al., 2025	⚪	⚪	🟢	🔴	🔴	🟢	🟢	🟡	🟡	🔴	🟢	High
Pollock et al., 2023	⚪	⚪	🟢	🟡	🟡	🟢	🟢	🟢	🟢	🟢	🟡	Moderate
Lin et al., 2016	⚪	⚪	🟢	🟡	🟡	🟢	🟢	🟡	🟢	🟢	🟢	Moderate
Russo et al., 2025	⚪	⚪	🟢	🟢	🟡	🟢	🟢	🟡	🟢	🟢	🟢	Moderate
Wark et al, 2024	🟢	🟢	🟢	🟡	🟡	🟢	🟢	🟡	🟡	🟢	🟢	Moderate
Anderson et al, 2022	🟢	🟢	🟢	🟡	🟡	🟢	🟢	🟡	🟡	🟡	🟢	Moderate

Risk of bias assessment based on Joanna Briggs Institute (JBI) critical appraisal tool for the Cohort Studies.

Q1. Were the two groups similar and recruited from the same population?
Q2. Were the exposures measured similarly to assign people to both exposed and unexposed groups?
Q3. Was the exposure measured in a valid and reliable way?
Q4. Were confounding factors identified?
Q5. Were strategies to deal with confounding factors stated?
Q6. Were the groups/participants free of the outcome at the start of the study (or at the moment of exposure)?
Q7. Were the outcomes measured in a valid and reliable way?
Q8. Was the follow up time reported and sufficient to be long enough for outcomes to occur?
Q9. Was follow up complete, and if not, were the reasons to loss to follow up described and explored?
Q10. Were strategies to address incomplete follow up utilized?
Q11. Was appropriate statistical analysis used?

Legend:

Yes

No

Unclear

Not applicable
